# Body composition in children with juvenile idiopathic arthritis: effect of dietary intake of macronutrient: results from a cross sectional study

**DOI:** 10.11604/pamj.2015.20.244.4488

**Published:** 2015-03-13

**Authors:** Asmae Hari, Samira Rostom, Asmae Hassani, Dalal El Badri, Ilham Bouaadi, Amina Barakat, Bouchra Chkirat, Khalid Elkari, Bouchra Amine, Najia Hajjaj-Hassouni

**Affiliations:** 1 Department of Rheumatology, El Ayachi Hospital, University Hospital of Rabat-Sale, Sale, Morocco; 2 Department of Pediatrics, Hospital of Children, University Hospital of Rabat-Sale, Rabat, Morocco; 3 Department of Nutrition, University Ibn Tofaïl, Faculty of Science of Kenitra, Kenitra, Morocco

**Keywords:** Juvenile idiopathic arthritis, macronutrients intake, lean body mass, fat mass, bone mineral content

## Abstract

**Introduction:**

The aim of this study was to evaluate the relationship between macronutrient intake, body composition (lean body mass and fat mass) and bone mineral content in Moroccan children with juvenile idiopathic arthritis (JIA).

**Methods:**

A cross-sectional study, conducted between May 2010 and June 2011, covering out patient with JIA. The characteristics of patients were collected. The nutritional status was assessed by a food questionnaire including data of food intake during 7 consecutive days using 24-hour dietary recall. Food intake was quantified using the software Bilnut (Bilnut version 2.01, 1991). Dietary intake of macronutrients was expressed as percentage contribution to total energy. Body composition was evaluated with DXA total-body measurements (bone mineral content BMC expressed in g, lean body mass LBM and fat mass FM expressed in kg).

**Results:**

33 patients were included. The mean age was 10.4 ± 4.3 years. The median disease duration was 2 (1-4.5) years. The median of LBM, FM and BMC were 19 kg (13.82-33.14), 5 kg (3.38-9.14) and 1044.90 g (630.40-1808.90) respectively. We found a positive correlation between LBM and dietary intake of carbohydrate (r= 0.4; p = 0.03). There were no significant association between LBM and intake of lipids, or protein. Moreover, no association was found between FM, BMC and intake of carbohydrates, lipids and proteins.

**Conclusion:**

This study suggests that there is a positive correlation between carbohydrates intake and LBM; however, dietary intake does not influence FM and BMC. Prospective studies with larger numbers of patients appear to be needed to confirm our findings.

## Introduction

In patients with juvenile idiopathic arthritis (JIA), growth impairment and altered body composition, including disturbed skeletal development, are well-known long-term complications [1]. Although, few studies have specifically addressed body composition in children with JIA.

Reduced bone mineral density (BMD) is also well recognized features [2]. Deficits of muscle mass have been described as a central factor in (secondary) bone loss [3]. The reasons for deficits in lean body mass are thought to be the disease activity itself, medication (corticosteroids), reduced physical activity, and malnutrition [4]. Apart from low BMD and lean mass, a higher fat mass was reported in children with rheumatic diseases [5].

Despite the considerable hereditary influence on body composition, the environmental factors, namely nutrition, play an important role as well [6]. Among the nutritional factors, macronutrients (proteins, carbohydrates and lipids), and their effects on bones receives most attention [7]. Inadequate dietary intake in childhood and adolescence might alter body composition with adverse implications later in life (obesity and osteoporosis). Therefore, understanding the effects of diet on body composition can guide the implementation of social policies and interventions that can help youths to develop healthy bodies.

In the literature, there was a very few studies assessing the relationship between dietary intake and body composition in children with JIA. In addition, there are no studies in a Moroccan population that evaluate the same subject. An abnormal body composition with low muscle mass and increased fat mass in children with JIA [4, 5] prompted us to conduct this study which aims to evaluate the relationship between dietary intake of macronutrients, body composition (lean body mass and fat mass) and bone mineral content in Moroccan children with JIA.

## Methods

### Data collection

A cross sectional study of children with JIA was conducted between May 2010 and June 2011 at the department of rheumatology of El Ayachi university hospital and department of pediatrics of university hospital of children of Rabat-Sale. Informed consent was obtained by parents from all subjects and the study was approved by ethics committee of our university hospital.

The diagnosis of JIA was based on the criteria of the International League of Association for Rheumatology (ILAR) [8]. Patients were recruited in consultation or during hospitalization. We excluded patients with any other chronic disease (endocrinal, neurological, cardiac, and renal) that affect bone metabolism. The disease and patients characteristics considered as explanatory measures were: age (year), gender, diagnosis (JIA subtype), disease duration (years). Disease activity was assessed using a visual analogical scale (VAS), number of tender joints, number of swollen joints, erythrocyte sedimentation rate (ESR), Disease activity score (DAS 28) for polyarticular and oligoarticular JIA [9]. The Maastricht AS Enthesitis Score and Bath AS Disease Activity Index (BASDAI) were used for juvenile spondylarthropathy [10]. Functional disability was determined by using the Moroccan version of Childhood Health Assessment Questionnaire (CHAQ) [11]. Treatment with NSAIDs, steroids and disease modifying anti-rheumatic drugs (DMARDs) was determined.

### Body composition

Body composition was evaluated with DXA total-body measurements (whole body bone mineral content BMC expressed in g, lean body mass LBM and fat mass FM expressed in kg) using the same DXA instrument (Lunar Prodigy; GE Lunar, Madison, WI). According to international consensus, DXA measurements without head were used. The instrument automatically alters scan depth depending on the thickness of the subject, as estimated from age, height, and weight. All scans were performed while the subjects were wearing light indoor clothing and no removable metal objects.

Medical history for bone fractures was negative in all patients. All subjects underwent plain Vertebral Fracture Assessment (VFA) to exclude unknown vertebral fractures [12].

### Dietary evaluation

Nutrient intake was determined using the 24 hour diet recall during 7 consecutive days [13]. The food questionnaire identified all foods consumed during the day previous to the interview. Two nutritionists analyzed the food dietary to quantify the food consumed from the recorded information. Nutrient intake was analyzed by software bilnut (Bilnut version 2.01, 1991), validated and standardized. Dietary intake of macronutrients was expressed as percentage contribution to total energy. None of the children was taking vitamin or mineral supplements at the time of recruitment

### Anthropometric measures

Weight (kg) and height (m) were measured according to the recommendation of the World Health Organization (WHO). The results of the BMI (Kg/m^2^) were compared with reference values of Hammer and al [14].

### Statistics

Analysis was carried out using the statistical package for the social sciences (SPSS) version 16.0. Data for patients were presented as mean ± standard deviation or median and quartile for continuous variables and as frequencies and percentage for categorical variables. The two-sample t-test was used for comparisons of scores within categorical variable subgroups. Pearson's linear correlation was used for the quantification between the observed numerical variables. Significance level was p value less than 0.05.

## Results

Thirty three patients were included in this study. The mean age of our patients was 10.4±4.35. 54.5% of our patients were males. The median disease duration was equal to 2(1-4.5) years. Demographic and clinical characteristics of patients are shown in Table 1. The median of lean body mass (LBM), total body fat mass (FM) and bone mineral content (BMC) were 19 kg (13.82-33.14), 5 kg (3.38-9.14) and 1044.90 g (630.40-1808.90) respectively (Table 1).

**Table 1:** Demographic and clinical characteristics of patients

Characteristics of patients	n = 33
Age (years), mean ± DS	10.4 ± 4.3
Female sex, n (%) Duration of disease (year), mediane (IQ)	15(45.5) 2(1-4.5)
**BMI, n (%)**	
Obesity	5(15.2)
Normal	19(57.6)
Underweight	9(27.3)
**Subtype of JIA, n (%)**	
Systemic	8(24.2)
Oligoarticular	9(27.3)
Polyarticular	16(48.5)
**Medications used, n (%)**	
NSAID	26 (79)
Corticosteroids	19(58)
DMARDs	17(51.5)
LBM (kg), mediane (IQ)	19 (13.82-33.14)
FM (kg), mediane (IQ)	5 (3.38-9.14)
BMC (g), mediane (IQ)	1044.90 (630.40-1808.90)

BMI: body mass index; JIA: juvenile idiopathic arthritis; NSAIDs: non-steroidal anti-inflammatory drugs; DMARDs: disease modifying anti-rheumatic drugs; LBM: lean body mass index; FM: fat mass, BMC: bone mineral content

The dietary intake data are shown in Table 2. Mean values for diet composition indicated that carbohydrates provided 53% of energy, lipids provided 32.5%, and proteins provided 16.1%.

**Table 2:** Energy, macronutrient intakes

	Total sample (33)	Boys (18)	Girls (15)	*p*
Carbohydrates (% of energy)	53±17.3	53.18±14.7	52.5±20	0.9
Lipids (% of energy)	32,5±13.1	30.8±13.4	34.6±12,8	0,4
Protein (% of energy)	16,08±4.3	16±3.8	16.2±5	0.9

Results are presented in mean±SD; Differences were tested using t test

We found a positive correlation between LBM and carbohydrates intake (r= 0.4; p = 0.03). There were no significant association between LBM and intake of lipids or proteins. Also, no association was found between FM, BMC and dietary intake of carbohydrates, lipids and proteins (Table 3).

**Table 3:** Pearson's correlation coefficients between dietary intake and body composition variables

	LBM	FM	BMC
Carbohydrates (% of energy)	0.4+	0.3	0.3
Lipids (% of energy)	-0.2	-0.09	-0.2
Proteins (% of energy)	-0.1	-0.04	-0.1

+ p<0.05, LBM: lean body mass, FM: fat mass, BMC: bone mineral content

## Discussion

Our data show that there is a positive correlation between LBM and carbohydrates ingested (expressed as a proportion of energy intake) but not between LBM, lipids and proteins intake. In the study of Volek JS et al, they found that a carbohydrate-restricted diet resulted in a significant reduction in fat mass and increase in lean body mass, which may be partially mediated by the reduction in circulating insulin concentrations [15]. Chandler-Laney PC et al showed that maternal glucose concentration during pregnancy was positively associated with children′s lean mass and fat mass [16]. In the other study, Lean muscle mass was not associated with physical activity or dietary intakes [17].

Previous observational studies of dietary protein intake and body composition have shown mixed results. Protein intake was not associated with LM in cross-sectional studies [17, 18]. In the Health ABC Study cohort, they found an association between protein intake and changes in LM over 3 years of follow-up [19]. However, few studies have examined the effect of protein intake on LBM in children.

The problem of overweight and obesity is a growing public health concern affecting numerous countries. The amount of fat ingested has been implicated as a causal or facilitating factor in the deposition of body fat. Thus, it is proposed that dietary fat can directly or indirectly manipulate human adipose tissue. There is suggestion in the literature that protein intake, not fat intake, may be associated with the development of adiposity in childhood [20]. It has been proposed that a high protein intake during early childhood stimulates insulin-like growth factor I production, thereby triggering precocious adipocyte multiplication [21]. In a prospective case-control study, they found that fat intake predicted gain in percentage of body fat in both adolescent girls with type 1 diabetes and healthy control girls [22]. In the other study, they did not find any association between dietary protein intake and percentage body fat [23]. Dietary carbohydrate expressed as a percentage of energy intake is often inversely related to body fat, including in childhood obesity [24]. In this study of children with JIA, there was no association between FM and intake of macronutrients.

Despite the methodological limitations of the present study; sample size, cross-sectional and non-controlled design, this study was the first Moroccan study that assesses the relationship between dietary intake and body composition in children with JIA, but the cause-effect relationship remains to be determined.

## Conclusion

This study suggests that there is a positive correlation between carbohydrates intake and LBM; however, dietary intake does not influence FM and BMC. Furthermore, Controlled feeding studies will be required to clarify possible roles of dietary intake in body composition of children with JIA.

## References

[1] Bechtold S, Roth Horm Res J (2009). Natural history of growth and body composition in juvenile idiopathic arthritis. Horm Res..

[2] Cimaz R (2002). Osteoporosis in childhood rheumatic disease: prevention and therapy. Best Pract Res Clin Rheumatol..

[3] Bechtold S, Ripperger P, Dalla Pozza R, Schmidt H, Häfner R, Schwarz HP (2005). Musculoskeletal and functional muscle-bone analysis in children with rheumatic disease using peripheral quantitative computed tomography. Osteoporos Int..

[4] Bardare M, Bianchi ML, Furia M, Gandolini GG, Cohen E, Montesano A (1991). Bone mineral metabolism in juvenile chronic arthritis: the influence of steroids. Clin Exp Rheumatol..

[5] Roth J, Palm C, Scheunemann I, Ranke MB, Schweizer R, Dannecker GE (2004). Musculoskeletal abnormalities of the forearm in patients with juvenile idiopathic arthritis relate mainly to bone geometry. Arthritis Rheum..

[6] Mul D, van Suijlekom-Smit LW, ten Cate R, Bekkering WP, de Muinck Keizer-Schrama SM (2002). Bone mineral density and body composition and influencing factors in children with rheumatic diseases treated with corticosteroids. J Pediatr Endocrinol Metab.

[7] Jasminka Ilich Z, Mario Skugor, Thomas Hangartner, An Baoshe, Velimir Matkovic (1998). Relation of Nutrition, Body Composition and Physical Activity to Skeletal Development: a Cross-Sectional Study in Preadolescent Females. Journal of the American College of Nutrition.

[8] Petty RE, Southwood TR, Manners P, Baum J, Glass DN, Goldenberg J, He X, Maldonado-Cocco J, Orozco-Alcala J, Prieur AM, Suarez-Almazor ME, Woo P (2004). International League of Associations for Rheumatology classification of juvenile idiopathic arthritis Edmonton, 2001. J Rheumatol..

[9] Ringold S, Yun Chon, Nora Singer G (2009). Associations between the American College of Rheumatology Pediatric Response Measures and the Continuous Measures of Disease Activity Used in Adult Rheumatoid Arthritis. ARTHRITIS & RHEUMATISM..

[10] Viswanath V, Myles A, Dayal R, Aggarwal A (2011). Levels of Serum Matrix Metalloproteinase-3 Correlate with Disease Activity in the Enthesitis-related Arthritis Category of Juvenile Idiopathic Arthritis. J Rheumatol..

[11] Rostom S, Amine B, Bensabbah R, Chkirat B, Abouqal R, Hajjaj-Hassouni N (2010). Psychometric properties evaluation of the childhood health assessment questionnaire (CHAQ) in Moroccan juvenile idiopathic arthritis. Rheumatol Int..

[12] Mäyränpää MK, Helenius I, Valta H, Mäyränpää MI, Toiviainen-Salo S, Mäkitie O (2007). Bone densitometry in the diagnosis of vertebral fractures in children: accuracy of vertebral fracture assessment. Bone..

[13] El Kari K, Borghos L, Benajiba N, Chabir R, Janah K, Schlossman N, Mokhtar N, Aguenaou H (2004). Daily vitamin A intake and nutritional disorders in preschool children: case of the northwest area of morocco. Report of the XXII International Vitamin A Consultative Group Meeting T38.

[14] Hammer DL, Kraemer HC, Wilson DM, Dornbush SM, Ritter PL (1991). Standardized percentile curves of body-mass-index for children and adolescents. Am J Clin Nutr..

[15] Volek JS, Sharman MJ, Love DM, Avery NG, Gómez AL, Scheett TP, Kraemer WJ (2002). Body composition and hormonal responses to a carbohydrate-restricted diet. Metabolism..

[16] Chandler-Laney PC, Bush NC, Rouse DJ, Mancuso MS, Gower BA (2011). Maternal glucose concentration during pregnancy predicts fat and lean mass of prepubertal offspring. Diabetes Care..

[17] Mitchell D, Haan MN, Steinberg FM, Visser M (2003). Body composition in the elderly: the influence of nutritional factors and physical activity. J Nutr Health Aging..

[18] Baumgartner RN, Waters DL, Gallagher D, Morley JE, Garry PJ (1999). Predictors of skeletal muscle mass in elderly men and women. Mech Aging Dev..

[19] Denise K Houston, Barbara Nicklas J, Jingzhong Ding, Tamara B Harris, Frances Tylavsky A, Anne Newman B, Jung Sun Lee, Nadine Sahyoun R, Marjolein Visser, Stephen Kritchevsky B (2008). (for the Health ABC study) Dietary protein intake is associated with lean mass change in older community-dwelling adults: the Health Aging and Body Composition (Health ABC) Study 1–3. Am J Clin Nutr..

[20] Rolland-Cachera MF, Davies PSW, Cole TJ (1995). Prediction of adult body composition from infant and child measurements. Body composition techniques in health and disease.

[21] Rolland-Cachera MF, Ulijaszek SJ, Johnston FE, Preece MA (1998). Adiposity rebound and prediction of adult fatness. The Cambridge encyclopedia of human growth and development..

[22] Särnblad S, Ekelund U, Aman J (2006). Diabetes Care. Dietary fat intake predicts 1-year change in body fat in adolescent girls with type 1 diabetes.

[23] Lisa-Marie Atkin, Peter SW Davies (2000). Diet composition and body composition in preschool children. Am J Clin Nutr..

[24] Tucker LA, Seljaas GT, Hager RL (1997). Body fat percentage of children varies according to their diet composition. J Am Diet Assoc..

